# Higher in-hospital proportion of breast milk intake improves brain functional connectivity and neurological assessment in preterm infants

**DOI:** 10.3389/fped.2025.1508840

**Published:** 2025-04-16

**Authors:** Rui Yang, Hua Wang, Qian Cai, Danqi Chen, Jiajun Zhu, Shuiqin Yuan, Fang Wang, Xinfen Xu

**Affiliations:** ^1^ School of Nursing, Capital Medical University, Beijing, China; ^2^ Women’s Hospital, School of Medicine, Zhejiang University, Hangzhou, China

**Keywords:** preterm infants, NICU, breast milk, neurological assessment, breastfeeding

## Abstract

**Objective:**

Preterm infants may face neurodevelopmental challenges linked to altered brain maturation processes. This study aimed to investigate the impact of in-hospital breast milk intake on brain resting-state functional connectivity (rs-FC) and neurological assessment at discharge in preterm infants.

**Methods:**

We collected data on breast milk intake from 97 preterm infants, evaluated neurological outcomes using the Amiel-Tison Neurological Assessment (ATNAT), and assessed rs-FC via functional near-infrared spectroscopy (fNIRS). Groups were stratified by breast milk intake proportion (cutoffs of >70% vs. ≤70%; cutoffs of >90% vs. ≤90%), and conducted logistic regression analysis to explore the relationship between rs-FC and neurological assessment.

**Results:**

Preterm infants with >70% breast milk intake exhibited significantly higher ATNAT levels ($\chi^{2}=\;8.306,\;$*p* = 0.004) and stronger rs-FC (*p* = 0.001) between the right precentral gyrus (PCG) and inferior parietal lobe (IPL). The >90% intake group also showed higher ATNAT levels ($\chi^{2}=\;7.090,\;$*p* = 0.008) and further rs-FC enhancements (PCG-PFL: *p* = 0.016; PCG-IPL: *p* = 0.008). Logistic regression confirmed rs-FC as a predictor of optimal neurological assessment [*p* = 0.011, Exp (B) = 0.206, 95% CI: 0.062– 0.682].

**Conclusion:**

Higher in-hospital breast milk intake (>70% of total enteral nutrition) improves rs-FC and neurological outcomes in preterm infants, with dose-dependent effects.

## Introduction

Approximately 15 million preterm infants are born annually worldwide, accounting for more than 1 in 10 infants (1). The incidence of preterm infants in China is approximately 7.0% (2). Preterm birth affects critical steps of third-trimester and early postnatal brain development, leading to a higher incidence of neurological disorders, including issues with cognition, language, motor skills, and social emotions (3–5). These infants have greater nutritional needs, and breastfeeding plays a crucial role in their neurological development (6, 7).

The duration of breastfeeding correlates positively with microstructural changes in brain (8, 9), particularly in regions such as the frontal and temporal lobes, the internal capsule, the corticospinal tract peripheral regions, the superior longitudinal fasciculus, and the superior fronto-occipital fasciculus. These areas are associated with higher cognitive functions, including executive function, planning, socio-emotional processing, and language (10). Breast milk is associated with improved structural connectivity of developing brain networks and greater fractional anisotropy in these major white matter fasciculi (10). Such structural improvements likely underlie the observed functional connectivity enhancements. Breast milk has the potential to enhance neurodevelopmental outcomes in preterm infants by promoting the healthy development of visual, language, motor, memory, higher cognitive, and emotional functions, thus improving overall neurodevelopmental outcomes. The benefits of breast milk on neurological development may persist into old age, with a particular emphasis on enhancing verbal reasoning abilities (11).

Due to the immaturity of various developmental aspects in preterm infants, their organ function and adaptability are generally inferior compared to term infants, necessitating that preterm infants receive specialized care in the neonatal intensive care unit (NICU) (12, 13). The majority of preterm infants in the NICU receive partial breastfeeding, which means breast milk constitutes a part of the nutritional intake for preterm infants in the NICU. Despite the well-established association between breast milk and the neurological development of preterm infants (14), the effects of early in-hospital breast milk intake volume on their neurological assessment remain unclear. Therefore, this study aimed to investigate the impact of in-hospital breast milk intake on brain resting-state functional connectivity (rs-FC) and neurological assessment in preterm infants. Functional Near-Infrared Spectroscopy was chosen to measure infants' FC for its suitability in neonatal neuroimaging, and Amiel-Tison neurological assessment for its validated reliability in assessing preterm neurological development. We hypothesized that infants with higher in-hospital breast milk intake would exhibit better neurological outcomes at discharge.

## Material and methods

### Participants

This observational study enrolled 106 preterm infants admitted to the neonatal intensive care unit (NICU) of a Grade III Level A hospital between December 2019 and February 2021. These infants had a gestational age of 30–34 weeks and Apgar scores ≥7 at 1 and 5 minutes. Exclusion criteria included congenital malformations, chromosomal abnormalities, moderate-to-severe hypoxic-ischemic encephalopathy, grade IV periventricular/intraventricular hemorrhage, cystic periventricular leukomalacia, congenital genetic metabolic diseases, and parental history of depression, mental illness, or congenital brain development disorders. Parental history of depression or mental illness was excluded to avoid confounding by heritable neurodevelopmental risks. Ethical approval was obtained from the hospital's ethics committee (approval number: 2019-058), and written informed consent was obtained from the parents. The study was registered as ChiCTR1900027648 on ClinicalTrials.gov and followed a previously published protocol (15). Due to COVID-19 pandemic-related home confinement and isolation policies, the recruitment period was extended, and the discharge follow-up section of the protocol was omitted.

### In-hospital breast milk intake

Mothers were encouraged to initiate breast milk expression immediately after delivery, with colostrum recommended for preterm infants as soon as available. Breast milk was the preferred nutritional source for preterm infants, provided the mother could express and deliver it to the NICU. Standardized feeding guidelines (16) were followed, supplemented with human milk fortifier (Similac HMFortifi, Abbott), once preterm infants reached enteral feed volumes of 80 ml/kg/day. In cases where parental consent was obtained for the use of donor human milk, it was utilized when the mother's own milk was insufficient, with preterm formula utilized as a last resort. For infants without parental consent, preterm formula was provided when maternal or donor human milk was insufficient. Daily nutritional intake in the NICU was documented electronically, encompassing both maternal and donor human milk. Detailed records of nutritional intake and volume were recorded daily, with the proportion of breast milk consumption calculated as the volume of breast milk intake relative to the total enteral nutrition volume during hospitalization.

### Neurological assessment at discharge

The neurological assessment of preterm infants was assessed at discharge by a trained NICU nurse using the Amiel-Tison neurological assessment (ATNAT). ATNAT is effective in detecting abnormal neurological signs, exhibiting good inter- and intra-assessor reliability and validity (17, 18). Consisting of 35 items, ATNAT utilizes a non-quantitative scoring system based on a three-point ordinal scale: “0” for typical responses, “1” for moderately abnormal responses, and “2” for definitely abnormal responses. Scores were assigned accordingly by the nurse, who remained blinded to infants' breast milk intake. The assessment typically takes 10–15 min to complete. To simplify analysis, responses categorized as “1” and “2” were combined to indicate non-optimal neurological assessment due to the infrequent occurrence of definitely abnormal responses (*n* = 2). Consequently, infants were classified as having either optimal or non-optimal neurological assessment.

### fNIRS measurements

Functional connectivity (FC) is investigated in early brain development to understand the functional integration of different brain regions (19). FC represents the statistical relationship between brain areas, forming functional connectivity networks (FCNs) (20). Functional Near-Infrared Spectroscopy (fNIRS) is a noninvasive neuroimaging technique sensitive to the developmental integration of circulatory, neurovascular, and metabolic functions in neonatal and infant brains (21). Previous studies have validated fNIRS for assessing the cerebrum (22), and recent studies had extended its utility to studying the neonatal cerebral cortex (23, 24).

Resting-state (rs) FC was measured using fNIRS at preterm infants' discharge. Infants were placed in a quiet, dimly lit room after feeding, where they wore fNIRS headgear. Data collection began once infants acclimated to the environment and headgear, with their actions recorded during the process. Soft silicone pads within the headgear and a headrest at the occipital lobe position aided measurements. Data collection occurred with infants wearing a electrocardiogram monitor, supervised by the same nurse.

**Figure 1:**
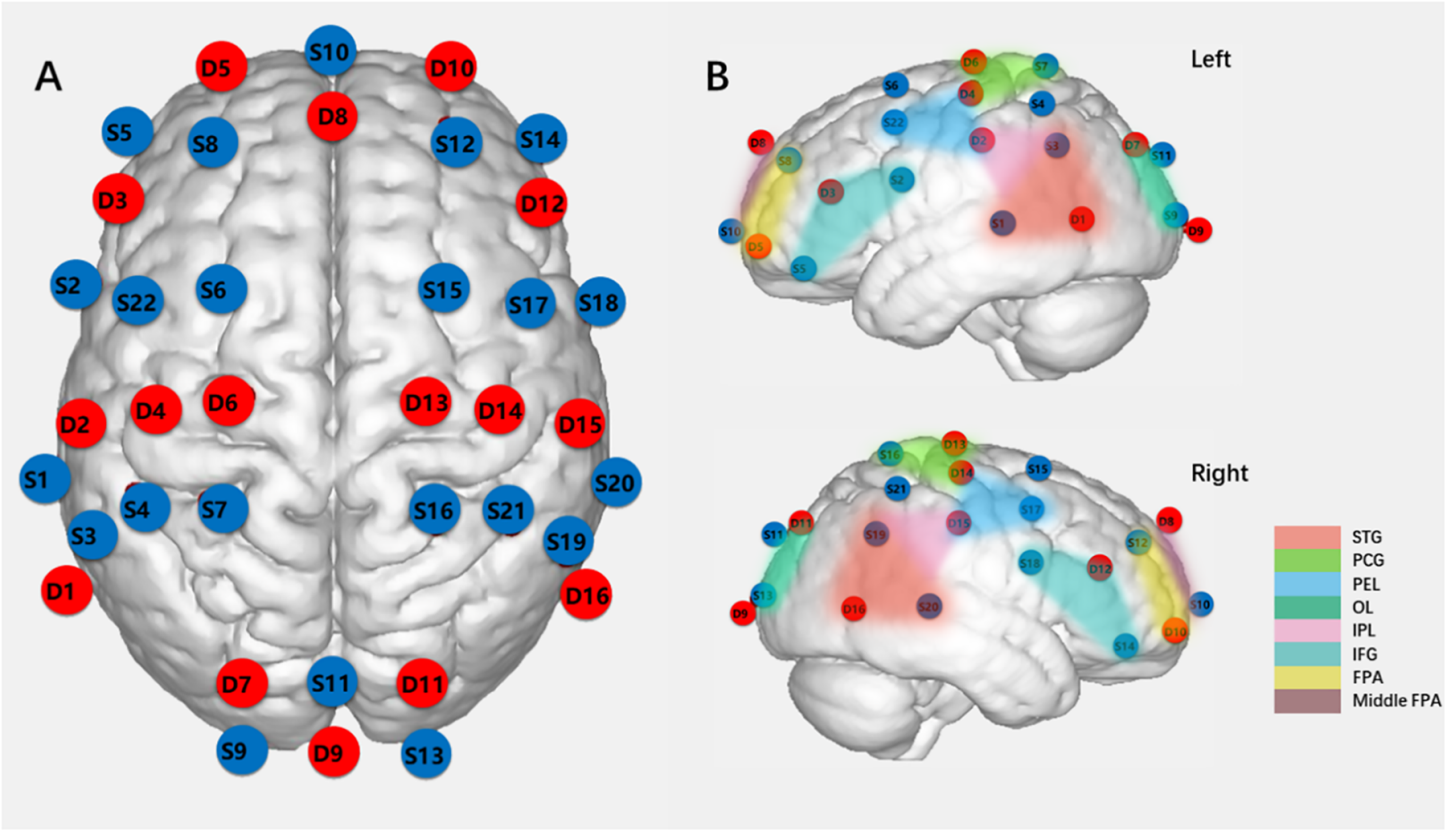
Schematic diagram of light sources and detection probes locations and ROIs (ROIs = 15). **(A)** Locations of 22 light sources and 16 dtection probes on fNIRs headcap (S:light source, D: dtection probe). **(B)** ROIs (Regions of interest), (STG:, superior temporal gyrus; PCG, precentral gyrus; PEL, posterior frontal lobe; OL, occipital lobe; IPL, inferior parietal lobe; IFG, inferior frontal gyrus; FPA, frontopolar area.).

Hemodynamic changes were collected using NirSmart (NirScan Inc., HuiChuang, Beijing), measuring oxygenated and deoxygenated hemoglobin concentration changes in the cortex at 760 and 850 nm wavelengths. The device, equipped with 58 channels (22 sources and 16 detection probes), had a sampling rate of 10 Hz. Optode probes were placed at T3, T4, Fpz, and Oz points following the international 10–20 system, with a 2.0 cm distance between the source and detector defining each measurement channel. The measurement area encompassed the prefrontal lobe, occipital lobe, bilateral motor area, and bilateral temporal lobe, comprising the regions of interest (ROIs) for this study. ROIs (*n* = 15) were determined based on Brodmann areas (25), as presented in Supplementary Table 1. Figure 1 illustrates the brain areas covered by the fNIRS array. Data were recorded using NIRScan software (NirScan Inc., HuiChuang, Beijing).

### fNIRS data processing

fNIRS data preprocessing was conducted using HomER2 (26), a MATLAB-based graphical user interface program. Channels with poor light intensity (signal-to-noise ratio <1.5), indicative of suboptimal optode-scalp contact, were excluded. Raw optical intensity was normalized to optical density (OD) by dividing by the mean intensity, yielding a relative (percent) concentration change. OD data were further converted to oxy-hemoglobin (HbO) and deoxy-hemoglobin (HbR) using the modified Beer–Lambert law (27).

During measurements, intervals corresponding to preterm infants' movements, crying, or other actions were manually marked as invalid by the same NICU nurse. Considering the minimum duration of −2 to 8 s required for infants' hemodynamic response function to return to baseline levels (28), 10 s of data were excluded after each invalid section across all channels to ensure the inclusion of only resting-state periods. Valid data sections were included only if they were consecutively at least 5 s long without interruption. Participants with ≥300 s of total valid data (29) and >70% reserved channels were included for further analysis. Artifact correction involved wavelet filtering (30) and principal component analysis (31), with a band-pass filter (0.01–0.1 Hz) applied to remove low-frequency noise and physiological interference.

### Statistical analysis

Demographic and clinical characteristics between groups were compared using the Mann–Whitney U test or two-sample t-test in SPSS 26.0. After obtaining FC values (Correlation, COR; Coherence, COH; Phase Locking Value, PLV) derived from hemodynamic signals (oxy-hemoglobin, HbO; deoxy-hemoglobin, HbR; total hemoglobin), between-group *t*-tests were conducted, with significant results corrected for multiple comparisons using the false discovery rate (FDR) method (32). COR reflects linear temporal relationships between hemodynamic signals (HbO, HbR and total hemoglobin) in distinct brain regions. COH measures frequency-dependent synchronization of signals, capturing shared oscillatory activity. PLV quantifies phase consistency between signals across trials, indicating stable inter-regional coupling. These metrics are widely validated in neonatal fNIRS studies (30–32). A 15 × 15 *p*-value matrix (COR of HbR) illustrating the results of between-group comparisons was generated.

Furthermore, multinomial logistic regression was performed in SPSS 26.0 to explore the relationships between FC and ATNAT scores, with confounding factors such as sex, mother's age, singleton status, delivery mode, gestational age, birth weight, and breast milk intake included as covariates. Covariates (sex, gestational age, etc.) were selected *a priori* based on their established impact on preterm outcomes (12, 15). A *p*-value of <0.05 was considered statistically significant.

## Results

### Demographic and clinical characteristics

**Figure 2:**
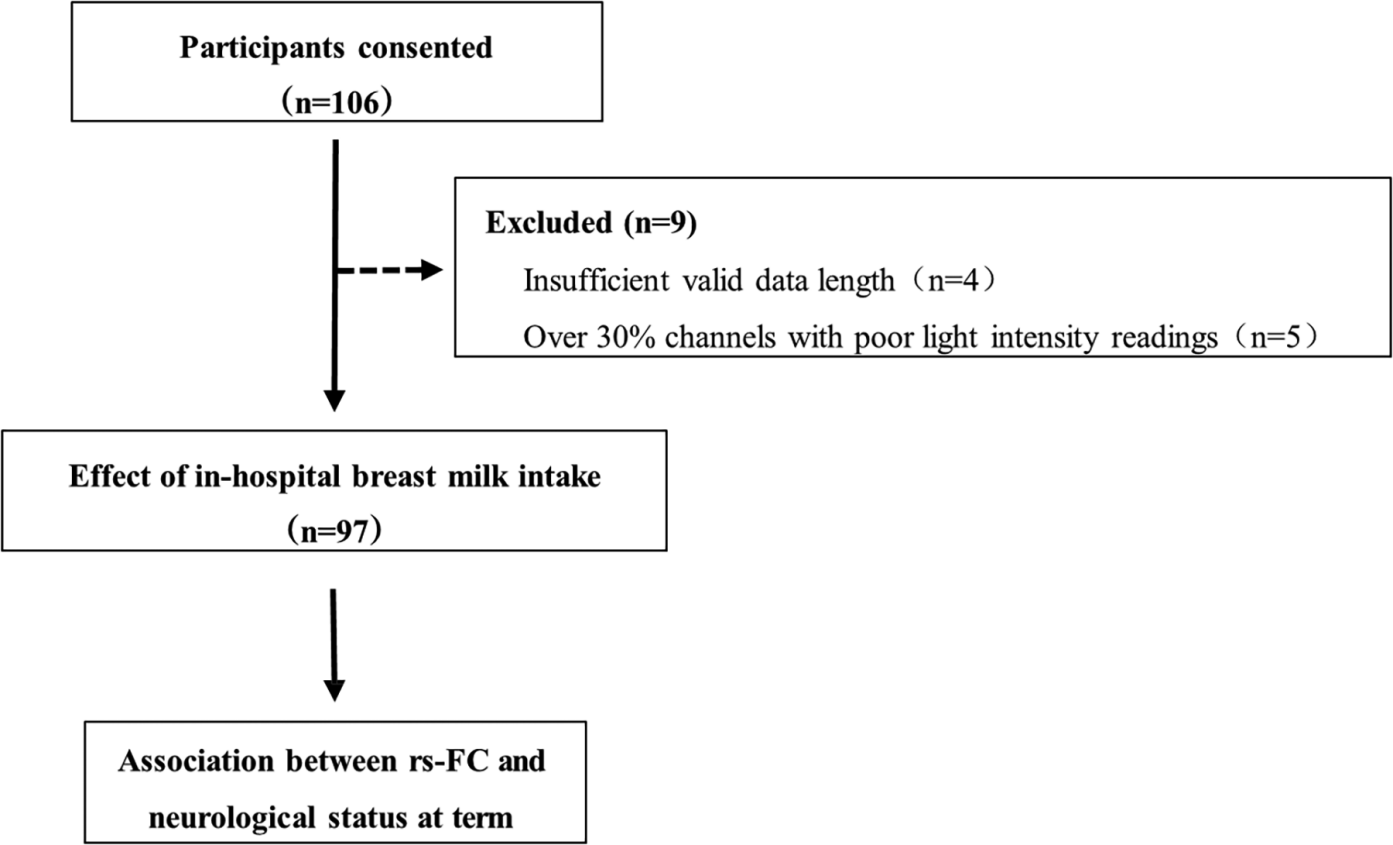
Participant flowchart. (rs-FC, resting-state functional connectivity).

A total of 106 participants were recruited for the study. Nine preterm infants were initially measured but subsequently excluded from the analyses due to insufficient valid data (<300 s), and five due to poor channel light intensity readings (>30%), as shown in Figure 2. Consequently, the study included 97 preterm infants, among whom 73 had breast milk intake exceeding 70% of in-hospital total enteral nutrition, while 24 had intake equal to or less than 70% (Table 1). Furthermore, subgroup analyses were conducted to assess the impact of breast milk intake, comparing infants with over 90% intake to those with 90% or less. Table 1 presents the demographic and clinical characteristics stratified by breast milk intake thresholds. The proportion of breast milk intake during hospitalization ranged from 5.73% to 100% for preterm infants.

**Table 1:** Demographic and clinical characteristics.

Variables	Breast milk intake volume reached 70%			Breast milk intake volume reached 90%		
	≤70% (*n* = 24)	>70% (*n* = 73)	*p*-value	≤90% (*n* = 32)	>90% (*n* = 65)	*p*-value
Gestational age, days	229.208 ± 7.852	228.644 ± 8.993	0.784	229.531 ± 7.100	228.415 ± 9.402	0.555
Birth weight, g	1,692.667 ± 301.482	1,637.671 ± 298.685	0.437	1,733.406 ± 320.388	1,610.846 ± 281.298	0.071
Female, *n* (%)	11 (46.8%)	43 (58.9%)	0.263	15 (46.9%)	26 (40.0%)	0.221
Mother's age, years	30.958 ± 3.223	31.164 ± 3.789	0.811	31.281 ± 3.540	31.038 ± 3.716	0.752
Singleton, *n* (%)	8 (33.3%)	38 (52.1%)	0.111	12 (37.5%)	34 (52.3%)	0.170
Cesarean section, *n* (%)	21 (87.5%)	62 (84.9%)	0.526	27 (84.4%)	56 (86.2%)	0.815
Apgar Score 1 min^a^	9.000 (1.250)	9.000 (0.000)	0.752	9.000 (2.000)	9.000 (1.000)	0.706
Apgar Score 5 min^a^	10.000 (0.000)	10.000 (0.000)	0.720	10.000 (0.000)	10.000 (0.000)	0.562
Age at measurement, days^a^^,^^b^	257.500 (7.000)	261.000 (8.000)	0.177	257.500 (7.000)	261.000 (8.000)	0.147
Weight at measurement, g	2,432.083 ± 406.233	2,359.452 ± 358.364	0.407	2,430.000 ± 290.244	2,351.538 ± 359.784	0.329

^a^ Median (interquartile range); Mann–Whitney *U*-test.

^b^ Age range, days (250, 280).

### Functional brain connectivity

**Figure 3:**
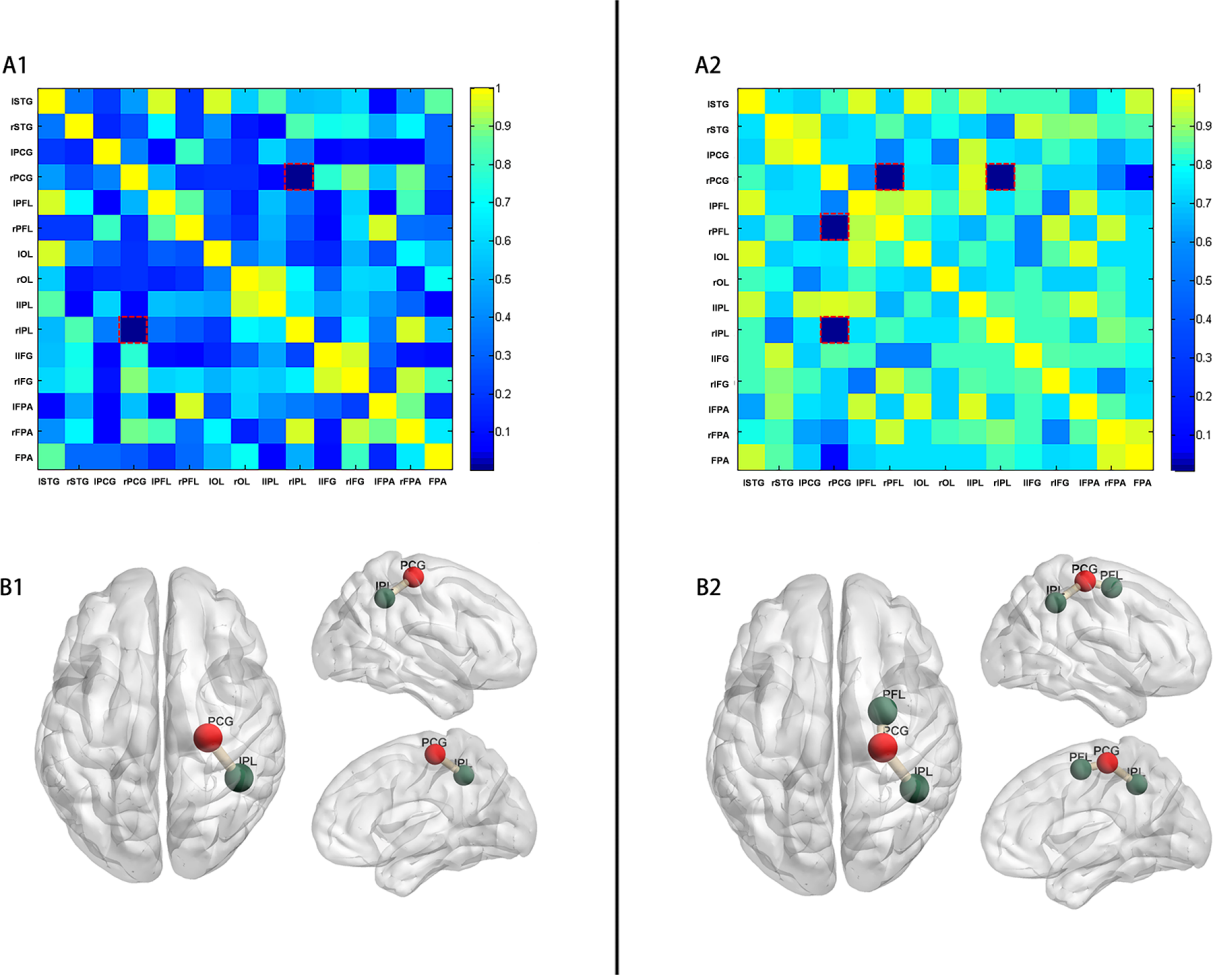
*p*-values of comparison of FC (COR of HbR). **(A)** *p*-values (adjusted *p*-values after FDR) matrix of t-test for FC (COR of HbR) between two groups of preterm infants (A1: preterm infants with in-hospital breast milk intake of >70% and ≤70%; A2: preterm infants with in-hospital breast milk intake of >90% and ≤90%). The horizontal and vertical coordinates represent the ROI number. **(B)** Resting-state FC was increased between the right PCG and the right IPL (*p* = 0.001) in preterm infants with in-hospital breast milk intake of >70% (B1). Resting-state FC was increased between the right PCG and the right IPL (*p* = 0.008) and between the right PCG and the right PFL (*p* = 0.016) in preterm infants with in-hospital breast milk intake of >90% (B2). (PCG, precentral gyrus; IPL, inferior parietal lobe; PFL, posterior frontal lobe).

FC between the right frontal lobe and right occipital lobe was significantly increased in preterm infants breastfed with >70% in the hospital (*p* = 0.001, FDR, Figure 3A1) compared to those breastfed with ≤70%. This connectivity involved the right precentral gyrus (PCG) and the right inferior parietal lobe (IPL), as illustrated in Figure 3B1. Furthermore, in preterm infants receiving >90% of in-hospital breast milk, FC in these neural systems was further enhanced, particularly in the right frontal lobe. Specifically, significant differences were observed in FC between the right PCG and right posterior frontal lobe (PFL) (*p* = 0.016, FDR, Figure 3A2), as well as between the right PCG and right IPL (*p* = 0.008, FDR). The anatomical labels depicting altered FC are presented in Figure 3B2.

### Neurological assessment at term

Significant differences were observed in the neurological assessment at discharge ($\chi^{2}=8.306,\;$*p* = 0.004) between preterm infants with >70% and those with ≤70% of in-hospital breast milk intake. A higher proportion of breast milk intake among preterm infants was associated with optimal neurological assessment at term age. Similarly, significant differences were noted in the neurological assessment at discharge ($\chi^{2}=7.090,\;$*p* = 0.008) between preterm infants with >90% and those with ≤90% of in-hospital breast milk intake volume.

Logistic regression analysis was conducted with demographic and clinical characteristics as covariates (sex, mother's age, singleton status, delivery mode, gestational age, birth weight, and breast milk intake), FC as a predictor, and ATNAT results as the outcome variable. The results are presented in Table 2. The regression model between FC (specifically, between the right PCG and right IPL) and ATNAT was statistically significant (*p* = 0.011, B = −1.578, Exp[B] = 0.206, 95% confidence interval [CI]: 0.062– 0.682; Hosmer and Lemeshow Test: Chi-square = 5.637, *p* = 0.688).

**Table 2:** Effects of ATNAT associated with FC in binary logistic regression models.

FC	Model	ATNAT at term equivalent age		
		OR	95%CI	*p*-values
rPCG- rIPL	Crude Model	−0.14^a^	−0.04∼−0.46	0.011
	Adjusted Model^b^	−0.24^a^	−0.06∼−1.05	0.010
rPCG- rPFL	Crude Model	−0.15^a^	−0.05∼−0.46	0.014
	Adjusted Model	−0.25	−0.07∼−0.93	0.066

^a^ *P*<0.05.

^b^ Adjusted model considered sex, mother's age, singleton, delivery mode, gestational age, birth weight and breast milk intake as covariates.

## Discussion

The study results indicate that preterm infants with >70% in-hospital breast milk intake exhibit better neurological assessment and enhanced development of rs-FC in the brain compared to those with ≤70% intake. Furthermore, our findings suggest that variations in rs-FC might correspond to differences in the neurological assessment of preterm infants. This study offers new insights into the neural basis of divergent neurological outcomes among preterm infants.

The present study unveiled an increase in rs-FC between the right PCG and the right supramarginal gyrus in preterm infants with an in-hospital breast milk intake volume exceeding 70%, with this positive effect demonstrating a dose-dependent pattern: infants with >90% breast milk intake exhibited stronger rs-FC than the >70% group, implying that higher proportions may yield incremental benefits. However, a definitive threshold requires validation in larger cohorts with granular intake data. Our findings support mother-infant bonding practices and DHM supply in NICU. These interventions may inhance infants' breast milk intake in NICU.

The PCG, serving as the primary motor cortex, and the supramarginal gyrus, situated in the IPL, jointly contribute to motor complexity processing, crucial for action perception and execution (33). Notably, previous research has highlighted a positive correlation between enhanced fine motor skills and enlarged PCG size in children (34). Furthermore, stroke patients exhibited improved upper limb movement following training, associated with increased FC of both the primary motor cortex and supramarginal gyrus (35).

The PFL encompasses the lateral and medial divisions of the pre-motor cortex and supplementary motor cortex. Alterations in rs-FC of the motor area have been observed in preterm infants, with a prevalent decrease in FC of the pre-motor cortex noted in very preterm infants compared to full-term counterparts (36). The development of executive functions, indicative of meaningful outcomes, has garnered attention (37). The observed increase in FC implies an enhancement in executive function related to volitional movement, suggesting that a higher proportion of early breast milk intake might contribute to improved executive function in preterm infants.

Blesa et al. (8) demonstrated an augmented fractional anisotropy-weighted connectivity, as observed through MRI, in infants who received ≥75% exclusive breast milk feeds compared to those who did not. Moreover, the degree of anatomical connectivity was further enhanced in these neural systems among infants who received ≥90% exclusive breast milk. Our results also indicate that increased functional connectivity (FC) serves as a predictor of the neurological assessment of preterm infants, independent of factors such as gestational age, birth weight, sex, singleton status, delivery mode, weight at assessment, and breast milk intake. This suggests that a higher proportion of breast milk intake might influence the neurological assessment of preterm infants by enhancing rs-FC. Preterm infants exhibiting increased FC were more likely to be assessed with optimal neurological assessment at their corrected gestational age at term. Evidence has suggested that early breast milk exposure may exert lasting effects on functional connectivity (38). For instance, longitudinal MRI studies report sustained improvements in white matter microstructure and cognitive outcomes in preterm infants receiving high breast milk volumes during hospitalization (10, 39). While our data are limited to the neonatal period, future studies should explore whether rs-FC enhancements observed here correlate with long-term neurodevelopmental trajectories.

Breast milk contains neuroprotective agents such as lactoferrin, insulin-like growth factor-1 (IGF-1), and long-chain polyunsaturated fatty acids (LC-PUFAs), which modulate neuroinflammation, synaptic plasticity, and myelination (14). Human milk oligosaccharides (HMOs) may further promote gut-brain axis signaling, indirectly supporting connectivity. Fortification with LC-PUFAs or HMOs could theoretically amplify these benefits, though evidence remains limited (40). A recent study found that breast milk was protective for preterm infants' emotionally reactiveand sleep problems (41), enhancing the positive effect of breast milk to brain development.

### Limitations

The present study has several limitations. The relatively small sample size may limit the generalizability of the findings. Additionally, the absence of normality tests for rs-FC connectivity values before conducting *t*-tests may have influenced the results. In future research, we intend to address these limitations by expanding the sample size and conducting comprehensive statistical tests to enhance the reliability and validity of our findings. This study focused on outcomes at discharge; however, whether rs-FC improvements persist beyond NICU care or correlate with long-term outcomes remains unknown. Future longitudinal studies should address this critical gap. While this study considered some confounding factors, completely eliminating them is challenging due to potential unobserved confounders or measurement errors. Therefore, while the use of covariates in this study may mitigate their impact, it cannot completely eliminate their influence.

## Conclusion

The study findings suggested that preterm infants with a higher in-hospital breast milk intake were more likely to demonstrate optimal neurological assessment compared to those with a lower intake. Moreover, this effect exhibits a volume-dependent relationship. Additionally, our results indicated that variations in rs-FC may correspond to differences in the neurological assessment of preterm infants.

## Data Availability

The raw data supporting the conclusions of this article will be made available by the authors, without undue reservation.
