# Supplementary material for: Higher in-hospital proportion of breast milk intake improves brain functional connectivity and neurological assessment in preterm infants
Source: Front Pediatr. 2025 Apr 16;13:1508840. doi: 10.3389/fped.2025.1508840 (PMC12040976; doi:10.3389/fped.2025.1508840)
Supplement: Supplementary file 1 [file Table1.docx]

**Supplementary Table 1.** Anatomical labels (Brodmann area) and ROIs associated with channels.

| Brodmann area | | Channels | ROI | Brodmann area | | Channels | ROI |
| --- | --- | --- | --- | --- | --- | --- | --- |
| 1. | Part of the superior temporal gyrus,  included in Wernicke's area | 1，5 | Left superior temporal gyrus (STG) | 2. | Part of the superior temporal gyrus,  included in Wernicke's area | 50,52 | Right STG |
| 3. | Primary motor cortex | 17,16 | Left precentral gyrus (PCG) | 4. | Primary motor cortex | 42,43 | Right PCG |
| 5. | Pre-Motor and supplementary  motor cortex | 12,13,56,58 | Left posterior frontal lobe (PFL) | 6. | Pre-Motor and supplementary  motor cortex | 39,40,45,46 | Right PFL |
| 7. | Primary and secondary visual cortex | 22,27 | Left occipital lobe (OL) | 8. | Primary and secondary visual cortex | 29,34 | Right OL |
| 9. | Supramarginal gyrus part of  Wernicke's area | 6 | Left inferior parietal lobe (IPL) | 10. | Supramarginal gyrus part of  Wernicke's area | 49 | Right IPL |
| 11. | Pars triangularis, part of Broca’s area | 4,10 | Left inferior frontal gyrus (IFG) | 12. | Pars triangularis, part of Broca's area | 36,37 | Right IFG |
| 13. | Frontopolar area | 20,24 | Left Frontopolar area (FPA) | 14. | Frontopolar area | 26,31 | Right FPA |
| 15. | Frontopolar area | 25 | Middle Frontopolar area (FPA) |  |  |  |  |
